# Landscape Movements of *Anopheles gambiae* Malaria Vector Mosquitoes in Rural Gambia

**DOI:** 10.1371/journal.pone.0068679

**Published:** 2013-07-18

**Authors:** Christopher J. Thomas, Dónall E. Cross, Claus Bøgh

**Affiliations:** 1 Institute of Biological, Environmental and Rural Sciences, Aberystwyth University, Aberystwyth, Wales, United Kingdom; 2 The Sumba Foundation, Kuta Poleng, Bali, Indonesia; Johns Hopkins University, United States of America

## Abstract

**Background:**

For malaria control in Africa it is crucial to characterise the dispersal of its most efficient vector, *Anopheles gambiae*, in order to target interventions and assess their impact spatially. Our study is, we believe, the first to present a statistical model of dispersal probability against distance from breeding habitat to human settlements for this important disease vector.

**Methods/Principal Findings:**

We undertook post-hoc analyses of mosquito catches made in The Gambia to derive statistical dispersal functions for *An. gambiae sensu lato* collected in 48 villages at varying distances to alluvial larval habitat along the River Gambia. The proportion dispersing declined exponentially with distance, and we estimated that 90% of movements were within 1.7 km. Although a ‘heavy-tailed’ distribution is considered biologically more plausible due to active dispersal by mosquitoes seeking blood meals, there was no statistical basis for choosing it over a negative exponential distribution. Using a simple random walk model with daily survival and movements previously recorded in Burkina Faso, we were able to reproduce the dispersal probabilities observed in The Gambia.

**Conclusions/Significance:**

Our results provide an important quantification of the probability of *An. gambiae* s.l. dispersal in a rural African setting typical of many parts of the continent. However, dispersal will be landscape specific and in order to generalise to other spatial configurations of habitat and hosts it will be necessary to produce tractable models of mosquito movements for operational use. We show that simple random walk models have potential. Consequently, there is a pressing need for new empirical studies of *An. gambiae* survival and movements in different settings to drive this development.

## Introduction

Knowledge of the movements (dispersal) of female vector mosquitoes between water bodies (emergence and subsequent egg laying) and human hosts (for blood meals) is fundamental to understanding spatial variation in malaria transmission rates [1] and specifically for planning and evaluating the impact of vector control [2]–[4]. The species complex *Anopheles gambiae sensu lato* contains some of Africa’s most efficient vectors of human malaria [5] yet, despite decades of effort [6], knowledge of their local dispersal remains sparse and inadequate. In the present study, we conducted a retrospective analysis of mosquitoes sampled in villages found at a range of distances from mapped larval habitat in The Gambia [7]. This geographic approach allowed us to statistically estimate the probability of female *An.gambiae* s.l. dispersing a given Euclidean distance between breeding sites and villages, thus quantifying an important parameter for spatial approaches to malaria control in this kind of landscape. We then use a very simple theoretical dispersal model to replicate this distribution.

There are two main approaches to estimating dispersal [8]. The first uses direct measurements of movements among locations by mark-release-recapture (MRR) experiments. For *An.gambiae*, such experiments are often limited by low recapture rates [9]–[11], and the difficulties in marking sufficiently large proportions of the population to have a chance of recording long-distance movements. In the second approach, observations are compared with those expected from a model. Empirical models, based upon expected frequency curves such as an exponential decay function with distance from source, may be estimated by sampling the numbers of individuals dispersing known distances [12]. Negative exponential decay functions of declining dispersal probability with increasing distance are generally suitable for modelling passive dispersal, but they may underestimate long-distance dispersal events in actively dispersing organisms [13], such as mosquitoes, that will keep moving until they satisfy the objective of their search. Although relatively rare, these longer movements may be of ecological importance [14], in this case for both malaria transmission and gene flow among populations of vector and parasite. Models using ‘heavy-tailed’ distributions, such as the half-Cauchy distribution [13], in which the rate of decline of probability of dispersal against increasing distance occurs at a slower rate than the negative exponential, are more consistent with the process of active, goal-directed dispersal in animals. Some of the best information on such dispersal of individual animals in wild populations comes from bird-banding, especially for the UK where an organised scheme has run since 1909. Here half-Cauchy distributions best described dispersal distances recorded for two UK bird species with very large samples marked and recaptured [8]. Process-based mechanistic models require an understanding of biological, geographical and physical determinants of movements across landscapes, for example the passive wind dispersal of plant propagules [15], but these parameters are insufficiently known for *An.gambiae* [4], [6] and are likely to be numerous, complex (e.g. [16]) and certainly difficult to measure. An alternative approach is to generate dispersal paths using simple theoretical movement rules, such as random walk models [17].

Heterogeneity in mean adult female mosquito density in villages is expected to be a function of distance to breeding sites [18], [19]. In most settings the distance travelled by mosquitoes entering villages would be unknown because of widely scattered, often unmapped, breeding sites. However, successive studies in our study area have demonstrated that most *An.gambiae* larval habitat is found on the periphery of alluvial sediments bordering the floodplain of the River Gambia [20]–[23], with villages found at varying distances from this edge, thus presenting an unusual opportunity to investigate the problem. In our analysis we made the simplifying assumption that the shortest Euclidean distance from villages to this habitat gave an estimate of functional mosquito dispersal distance. Note, this is not the length of the route travelled by mosquitoes, which depends on flight path and is unknown. We calculated both negative exponential and half-Cauchy probability density functions (PDF) and cumulative probability of dispersal against distance.

The majority of movement by female mosquitoes seeking blood meals appears not to be in response to host cues: the only field experiment on freshwater *Anopheles* of which we are aware suggests that orientation towards a host could account only for short range movement over 30 m or less [24]. However, there is little consensus on the movement of mosquitoes prior to finding host odour plumes [25]. An early field experiment suggested directed movement towards human habitation [26] although more recent studies suggest random movements [27], [28]. Uncorrelated random walk diffusion results in a Gaussian distribution with exponential decay in probability of dispersal against distance, but scale is determined by step-length and survival/step. We simulated dispersal with a simple uncorrelated random walk model, using minimum daily survival and movement length estimated from a 1996 MRR study in a similar rural landscape in Burkina Faso [9] and compared the results to the statistical distribution we observed in The Gambia. The minimum daily survival rate in Burkina Faso was very similar to Gilles’ 1961 estimate from MRR experiments in east Africa [26].

## Methods

We conducted a post-hoc reanalysis of data presented in our earlier study in The Gambia, using mosquito catches made in villages in 1996 [7].

### Location

**Figure 1:**
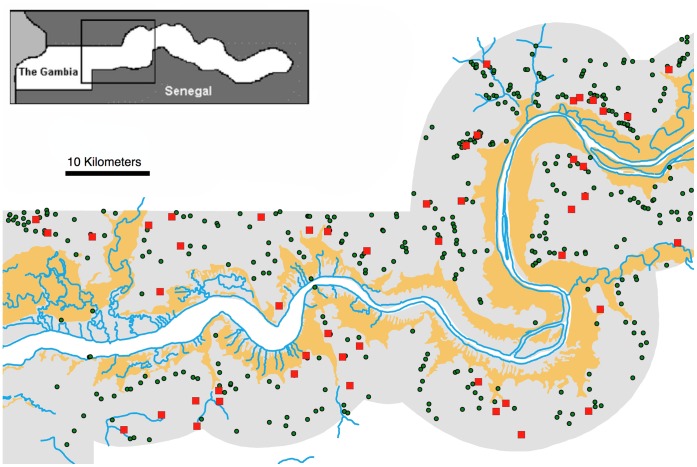
Map of the study area. Central Gambia, showing study villages (red squares), other villages (green circles), main channel of The River Gambia and tributaries (blue lines), alluvial sediment (solid yellow). The Gambia nation is shaded grey, surrounded by Senegal in white. Inset: location of the study area in The Gambia.

The 1996 study was conducted in central Gambia (Figure 1), a rural area dominated by secondary bush and land cleared for agriculture with widely spaced discrete villages [20], [29], typical of the West Sudan Savanna ecotype (World Wildlife Fund, terrestrial ecoregion AT0722). 48 study villages were selected by geographically stratified random selection by dividing the study area into 8 sectors of approximately equal size and randomly selecting 6 villages in each.

### Entomology

Mosquitoes were collected in bi-weekly, one-night catches (1900–0700 hrs) using two CDC miniature light traps in each of the study villages, situated in bedrooms of single occupants living in the part of the village closest to the river, during the wet season (July-Dec) of 1996. Mosquitoes were sexed and species identified in the laboratory by morphology from which the geometric mean (GM) number of female *An. gambiae* s.l./night was calculated [7]. Only GM values were available to the present study.

### Mapping

Study villages were identified on a GIS (ArcGIS10) coverage of all settlements digitized from contemporary 1∶50,000 scale maps [30] in UTM projection, updated using 1993 census maps from the Gambian Central Statistics Department [31]. The landward border of alluvial sediments forming the floodplain of the River Gambia was defined using a digital GIS coverage of soils derived from interpretation of 1982 aerial photos [32]. Land cover classes comprising saline, wet-seasonally flooded mudflats and barren tidal flats were combined to define alluvial sediment containing *An. gambiae* breeding habitat within the study area [20], [21] (Figure 1). The remaining riparian habitat, mangrove, was excluded as previous studies had found no *An.gambiae* larvae in this habitat [20], [21]. Satellite imagery was used to confirm that no changes had taken place in the location of the landward border of alluvial sediments since mapping. A moistness index was derived using a Kauth and Thomas tasseled cap 4-dimensional transformation using Idrisi 32 image processing software on bands 1–5 and 7 of a Landsat 5 TM image, acquired on 28 November 1990 (the most contemporary archived image available). GIS overlay of the soils coverage with this index confirmed that the 1982 coverage still accurately delineated the alluvium boundary in 1990, and there is no reason to suspect any subsequent changes had occurred by 1996.

### Analysis

The distance from each village to the nearest *Anopheles* larval habitat was computed as the shortest Euclidean distance from village edge to the landward border of the alluvial sediment. Values for GM female *An.gambiae* s.l/night in each of the 48 villages were not significantly different from that expected under a normal distribution (One-Sample Kolmogorov-Smirnov Test, Z = 1.338, p = 0.056) so untransformed values were used in subsequent analyses. Non-linear regression was used to model the GM *An.gambiae* s.l./night against distance from the village to the nearest breeding habitat, using two-parameter negative exponential and half-Cauchy PDFs:
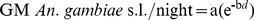
(1)

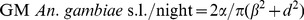
(2)where d is distance dispersed (km) and (a, b) and (α, β) are parameters estimated in regression models 1 and 2 respectively. Model residuals were tested for spatial autocorrelation using Moran’s I in 1 km distance classes for 0–10 km, measuring symmetric distances between pairs of points, with significance tested against 999 permutations. The cumulative predicted proportion of mosquitoes moving a given distance was plotted within a maximum range of 10 km. Statistical analyses were performed using Matlab vR2012b and the Moran’s I correlogram with SAM v4 [33].

### Simulation

A particle-tracking random walk model with discrete time and continuous space was used to distribute 10,000 mosquitoes (particles) from a single start location in daily time steps, with each mosquito moving in a randomly-chosen direction for a given daily step length, whereupon direction was reassigned (independently of previous movements) and a subsequent movement made [17]. At each step a number of mosquitoes was randomly selected and withdrawn from the pool moving (simulating death), based on the daily survival probability. The Euclidean distance from source to position at death was recorded. The simulation continued until the population was extinct or 30 days whichever came first, whereupon the proportions moving distances in 100 m classes was calculated. The simulations were made using a survival rate of 0.8/day and movement rate of 350 m/day [9] repeated 1000 times.

### Ethics Statement

Ethical approval for the original 1996 field study was obtained from the joint Gambian Government/UK Medical Research Council Ethical Committee. Informed consent was obtained from village leaders, the heads of family compounds and households selected for mosquito trapping.

## Results

**Figure 2:**
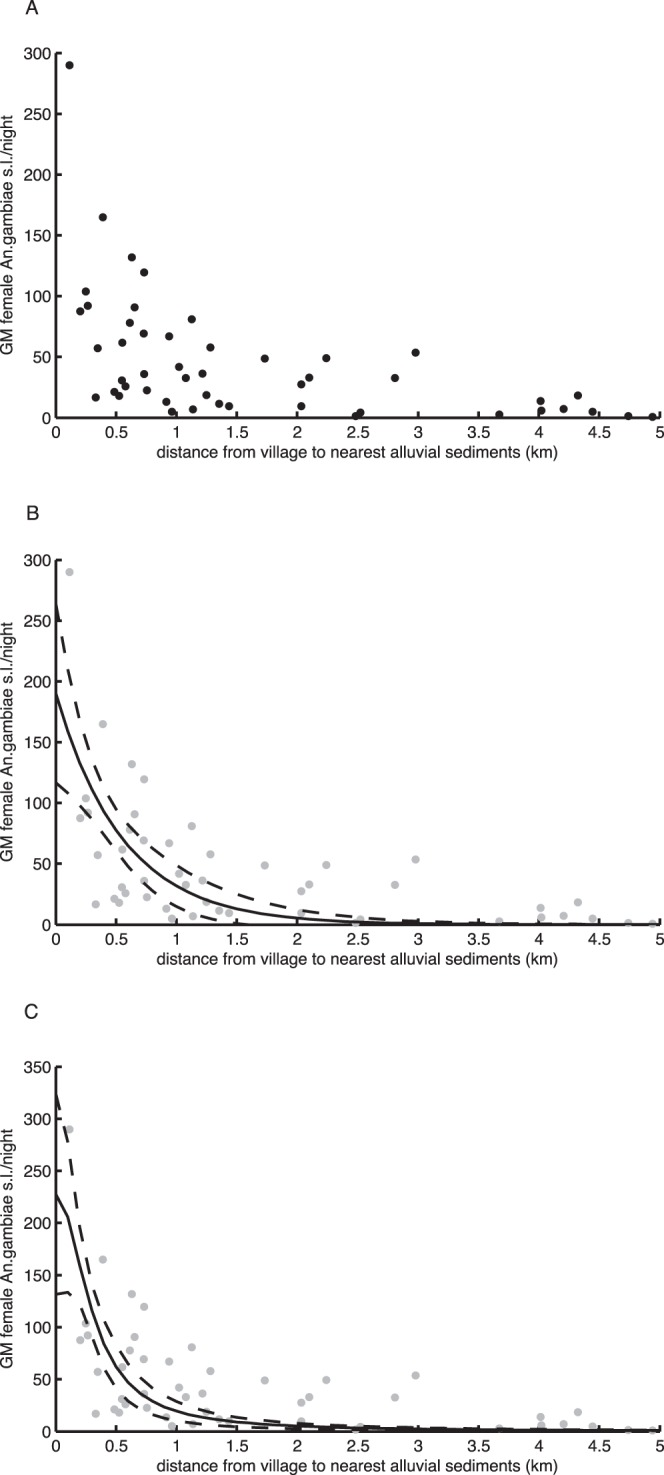
Mosquito dispersal in The Gambia. A) Geometric mean (GM) female *Anopheles gambiae* s.l./night in villages in the rainy season (Jul–Dec) 1996 at different distances from the landward edge of alluvial sediments; B) Two-parameter negative exponential non-linear regression model (solid line) with 95% CIs of curve (dashed lines) with points from A in grey for reference; C) Two-parameter half-Cauchy non-linear regression model (solid line) with 95% CIs of the curve (dashed lines) with points from A) in grey for reference.

The 48 villages ranged from 115–4943 m from the border of alluvial sediments; equal numbers of villages were located north and south of the river. A total of 1,043 light trap catches were carried out, yielding 65,024 individual female *An.gambiae* s.l. GM female *An.gambiae* s.l. in study villages ranged from 0.8–289.1 mosquitoes/night [7]. The number of mosquitoes caught declined steeply with distance to nearest breeding habitat (Figure 2a). There was no effect of being on the north or south bank of the river on dispersal distance vs. mosquito numbers (ANCOVA ln(GM) vs. distance; interaction bank*distance, F = 2.55, p = 0.118).

**Figure 3:**
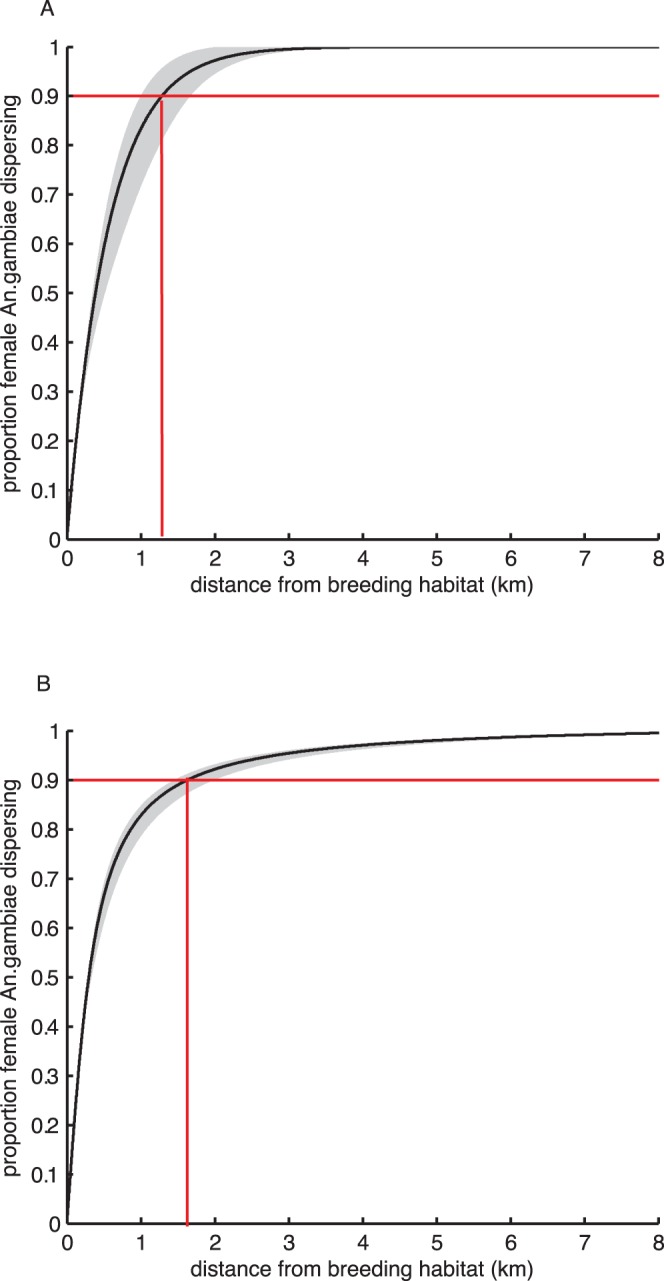
Probability of dispersal. Cumulative probability of female *Anopheles gambiae* s.l. dispersal into villages in The Gambia versus distance from village to nearest alluvial sediments (solid line) estimated from non-linear regression. Grey shaded areas indicate 95% CI of the curve and red marker lines indicate distances over which 95% of the population has dispersed; A) two parameter negative exponential model; B) two-parameter half-Cauchy model.

Non-linear regression produced a highly significant negative exponential model (a = 190.250, SE_a_ = 2.862; b = −0.172, SE_b_ = 0.037; F = 32.9, p<0.001), in which distance from the breeding sites explained over 40% of the variance in GM female *Anopheles gambiae* s.l. trapped in villages (Figure 3a; R^2^ = 0.417). The half-Cauchy model was also highly significant (α = 10.627, SE_α_ = 9.007; β = −0.306, SE_β_ = 0.066; F_1_ = 47.86, p<0.001) explaining a similar amount of the variance (Figure 3b; R^2^ = 0.417). There was no spatial trend in model residuals evident from spatial autocorrelation analyses of residuals in 1 km distance separation classes (Tables S1 and S2) up to 7 km. There was no statistical support to select one model over the other from Akaike’s Information Criterion, corrected for sample size (negative exponential model AIC_c = _493.658; half-Cauchy model AIC_c = _493.646) [34].

**Figure 4:**
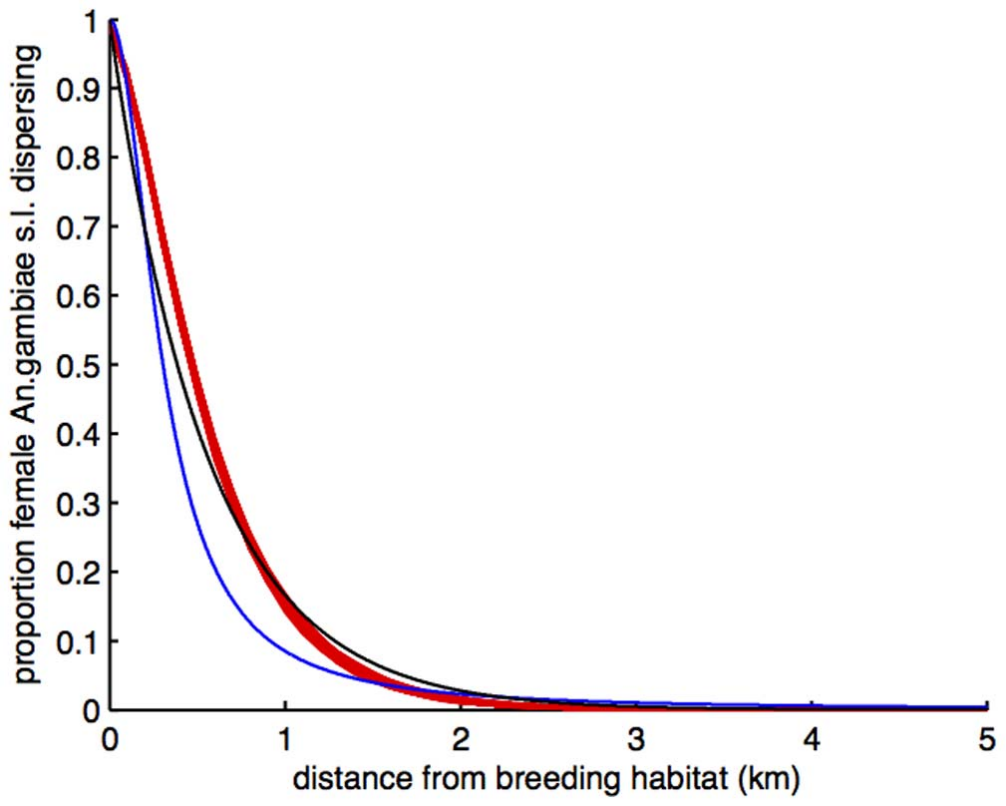
Random walk simulation. Two parameter negative exponential (blue line) and half-Cauchy (black line) regression models of female *Anopheles gambiae* s.l. dispersal into villages and simulated dispersal distances (red, 1000 simulations) from a random walk (daily survival = 0.8; daily movement = 0.35 km) of 10,000 mosquitoes emerging from a point source and dispersing until population extinction or day 30, whichever came first.

The cumulative percentage of *An. gambiae* s.l. adult females dispersing up to 10 km from breeding habitat is shown for 0.5 km distance bands up to 3 km in Table 1. Dispersal may be also be expressed as the distance moved by a given proportion of the population. Using the negative exponential function (Figure 3a) we predicted that 50% of *An. gambiae* adult females moved within 386 m from the nearest breeding site, 75% within 773 m, 90% within 1.284 km and 95% within 1.671 km. Using the half-Cauchy function (Figure 3b) these were predicted to be 50% within 295 m, 75% within 691 m, 90% within 1.635 km and 95% within 2.827 km. Simulation of 1000 random walk models each of 10,000 mosquitoes resulted in very similar dispersal probability curves (Figure 4).

**Table 1:** Predicted dispersal of adult female *Anopheles gambiae* s.l. mosquitoes away from breeding sites in The Gambia, using a 2-parameter negative exponential regression model and a 2-parameter half-Cauchy regression model.

	Proportion (%) of mosquitoes found within distance of larval habitat (95% CI of the curve)	
Distance from larval habitat (km)	Negative exponential	Half-Cauchy
0.5	59.2 (53.6–69.3)	66.4 (63.7–72.0)
1.0	83.3 (76.6–95.1)	82.7 (80.7–87.0)
1.5	93.2 (88.5–99.9)	88.9 (87.5–91.9)
2.0	97.2 (94.5–100)	92.1 (91.1–94.3)
2.5	98.9 (97.4–100)	94.1 (93.3–95.7)
3.0	99.5 (98.8–100)	95.4 (94.8–96.7)

## Discussion

We have used a novel approach to estimate for the first time the dispersal probability of female *An. gambiae* s.l. across a rural African landscape. This opportunity was afforded by the particular geographic layout of breeding sites and villages in central Gambia. Both negative exponential and half-Cauchy distributions fitted the decline in geometric mean numbers of female *An.gambiae* s.l. found in 48 villages ranging from adjacent to almost 5 km away from the border of breeding habitat. Both probability density functions were highly significant and fitted observed values equally well, and using information criterion there were no statistical grounds to select one model over the other. The dispersal of adult females of the Eastern Equine Encephalitis vector, *Culex erraticus*, has also been found to fit a negative exponential distribution with increasing distance to breeding sites [35], as have *Aedes aegypti* larvae numbers with distance to adult habitat in Rio de Janeiro [36]. However, the half-Cauchy function is considered the more biologically plausible distribution, due to evidence that such a heavy-tailed distribution may be a better model of active dispersal, for example in medflies *Ceratitis capitata* [37] and so is also included here. In his seminal work on *An. gambiae* in East Africa Gilles (1961) [26] found 4% of marked mosquitoes dispersed beyond 3.2 km: from the half-Cauchy model from The Gambia we predict 4.19% (95% CI 3.01–4.75%) moving greater than 3.2 km but for the negative exponential model only 0.32% (95% CI 0 - 0.87%), which suggests a heavy-tailed dispersal probability distribution.

Over 40% of the variance in mosquito numbers sampled in villages was explained by the Euclidean distance to the nearest likely breeding habitat. Much of the remaining variance is likely to be due to sources not included in our simple spatial model [35], [38]. Prime amongst these are unmapped breeding sites and the quality [21] and amount [7], [20], [23] of breeding habitat surrounding villages. It has also been suggested that differences in habitat matrix permeability caused by barriers to dispersal or gradients in suitability for dispersal in the landscape [39] may lead to geographic variation in functional distances. However, experiments by Gilles and colleagues [40] found no effect of artificial barriers on anopheline dispersal. The study area is one of low relief and relatively uniform habitat away from the river, so any permeability effect is unlikely to be large. There may also be shadow effects where villages away from the river have reduced encounter rates as mosquitoes curtail their dispersal in villages closer to breeding sites. For example, within a village in Tanzania, higher densities of mosquitoes were found in peripheral houses immediately adjacent to breeding sites [41], [42] and this has also been suggested in The Gambia [43]. However, any shadow effect was likely to be weak at the landscape scale considered here because villages in the study area tended to be compact and widely spaced relative to the extensive *An. gambiae* breeding habitat (Figure 1).

In addition to flight, mosquitoes may be carried passively on the wind over large distances [44], [45] and wind direction can influence local mosquito movement [18], [43]. South to south-westerly winds predominate in The Gambia, but the lack of evident difference between villages on North (mainly upwind of breeding sites) and South banks (mainly downwind) indicate that this was not a major factor over the distances considered here and supports the conclusion that mosquito dispersal is an active process at the landscape scale [6].

Our empirical dispersal-distance probability distributions for *An. gambiae* s.l. are remarkably similar to the simulated, simple random walk distribution using minimum mean survival (0.8/day) and movement rates (350 m/day) of *An.gambiae sensu stricto* and *An. arabiensis* estimated in Burkina Faso [9], from field MMR (Figure 4). Like our seasonally averaged capture data in relation to Euclidean distance to source, movements recorded by MMR are Euclidean distance from mark point to recapture point, not the true distance flown by mosquitoes (see [28]), but here in daily steps. The minimum daily survival and dispersal parameters obtained in Burkina Faso appear commensurate with observed dispersal in a similarly open landscape in The Gambia, despite the limitations associated with MRR [35]. This supports suggestions that pre-host-orientation dispersal is described by some form of random movement, although our data are not able to test for more complex (and biologically likely) patterns such as correlated random walks or Levi searches, as we were unable to discriminate statistically between exponential and heavy tailed dispersal. Landscape configuration will determine realised functional dispersal, as blood seeking mosquitoes curtail their movement on encountering a host. As Gillies and de Meillon noted 35 years ago [46], mosquito flight range is surely characteristic of environment. Analyses of *An. gambiae* gene flow amongst villages in Mali [47], indicated less movement than in Burkina Faso [9] where villages were closer. In urban environments, dispersal distances are usually considerably less than in rural areas, e.g. [38], [48]–[50]. How to metric the landscape geometry of breeding habitat in relation human hosts to estimate *Anopheles* dispersal remains a key question for future research. Landscape-scale hydrology can be used to estimate the spatial and temporal distribution of breeding habitat over landscape scales [51], [52].

What are the implications of our findings for malaria control? Considerable use is made of dispersal distance in applied control programs (e.g. [35], [47], [53]–[56] ), and in modelling the effects of such interventions (e.g. [57]). We consider briefly two widely used interventions; larval control and insecticide-treated nets. A drastic reduction in mosquito biting is needed to reduce transmission [3] and we have shown that small but not insignificant proportions of *An. gambiae* populations may disperse several kilometres from breeding sites (c. 8% beyond 2 km and c.5% beyond 3 km according to half-Cauchy distribution estimates; Table 1). In landscapes with extensive or widely scattered breeding sites, it will therefore be a considerable challenge to reduce mosquito numbers solely by larval control sufficiently to lower transmission and indeed this was the conclusion from such an attempt in central Gambia [23]. However, if breeding sites are localised, larval control within such a cordon sanitaire [58] may be a useful component of integrated malaria control [59]–[61]. Consideration of local geography and probability of *An.gambiae* dispersal into control areas is required to help inform such decisions. The dispersal ability of *An. gambiae* also has implications for the evaluation of insecticide treated bed-nets. As theoretical models [3] and field studies [11], [54] have indicated, the movement of mosquitoes can hide mass killing effects in paired village trials and consequently underestimate their community-wide effectiveness. Occasional exchange of *An. gambiae* has been found between villages 2 km apart in Mali [54], 3 km apart in Tanzania [10] and to a greater degree in The Gambia, in villages 1–1.4 km distant [11]. The *An.gambiae* s.l. dispersal-distance probability curves presented here demonstrate the likelihood of such inter-village exchange taking place in our study site (Table 1), assuming no philopatry, as multiple villages are usually within flight range of breeding sites. This supports the view [3] that in rural settings, large, contiguous areas should be the unit of assessment of these interventions, and certainly not individual settlements within 3 km. To target and assess interventions against the vector there is an urgent requirement for field measurements of *Anopheles* dispersal in different ecological settings, including urban, to drive a new generation of spatially explicit, tactical malaria transmission models that can incorporate landscape mediated vector dispersal. Spatially explicit, coupled larval and adult mosquito field surveys, combined with high resolution mapping of habitats, can be designed to test theoretical movement models. There is also considerable potential in the use of isotopes in MRR experiments [26], following the recent successful application of this technique to water bodies, thereby marking naturally-breeding mosquitoes at known sources [62].

## Supporting Information

Table S1 **Moran’s I spatial autocorrelation in residuals from a two-parameter negative exponential non-linear regression model of GM mosquitoes in villages versus distance from alluvial sediments.**
(DOCX)

Table S2 **Moran’s I spatial autocorrelation in residuals from a two-parameter half-Cauchy non-linear regression model of GM mosquitoes in villages versus distance from alluvial sediments.**
(DOCX)
