# Supplementary material for: Landscape Movements of Anopheles gambiae Malaria Vector Mosquitoes in Rural Gambia
Source: PLoS One. 2013 Jul 18;8(7):e68679. doi: 10.1371/journal.pone.0068679 (PMC3715529; doi:10.1371/journal.pone.0068679)
Supplement: Table S1 — Moran’s I spatial autocorrelation in residuals from a two-parameter negative exponential non-linear regression model of GM mosquitoes in villages versus distance from alluvial sediments. (DOCX) [file pone.0068679.s001.docx]

| Distance class | Count | Moran’s I | p |
| --- | --- | --- | --- |
| 1-1000 | 2 | -0.039 | 0.804 |
| 1001-2000 | 12 | 0.427 | 0.221 |
| 2001-3000 | 22 | 0.048 | 0.844 |
| 3001-4000 | 22 | -0.065 | 0.759 |
| 4001-5000 | 26 | -0.541 | 0.065 |
| 5001-6000 | 20 | 0.239 | 0.412 |
| 6001-7000 | 30 | -0.229 | 0.307 |
| 7001-8000 | 32 | -0.428 | 0.09 |
| 8001-9000 | 24 | -1.029 | 0.01 |
| 9001-10000 | 18 | 0.005 | 0.995 |
